# Chinese Migrant Adolescents’ Perceived Discrimination and Psychological Well-Being: The Moderating Roles of Group Identity and the Type of School

**DOI:** 10.1371/journal.pone.0146559

**Published:** 2016-01-05

**Authors:** Xia Liu, Jingxin Zhao

**Affiliations:** 1 Institute of Developmental Psychology, Beijing Normal University, Beijing, China; 2 School of Psychology, Shandong Normal University, Jinan, China; University of Illinois-Urbana Champaign, UNITED STATES

## Abstract

Perceived discrimination can be harmful to migrant adolescents in China. However, little is known about the processes through which discrimination may be linked to decreased well-being in Chinese migrant adolescents. This study examined the relationship between perceived discrimination and three indices of psychological well-being (self-esteem, life satisfaction, collective self-esteem) in 798 Chinese migrant adolescents (49.4% from public schools). Group identity affirmation and belonging (GIAB) was examined as a protective factor that was expected to alleviate the negative effects of perceived discrimination on well-being, and the type of school was investigated as a potential moderator of the associations of interest. The results indicate that perceived discrimination was negatively linked to the three indices of psychological well-being and that the negative effects of perceived discrimination on psychological well-being were particularly salient for migrant adolescents attending public schools. Additionally, GIAB emerged as a protective buffer against perceived discrimination’s negative effects on collective well-being.

## Introduction

Internal migration in mainland China is characterized by population flows from rural to urban areas, where individuals hope to find better living conditions. In recent years, the number of children migrating to cities with their parents has increased dramatically, as the pattern of migration has shifted from personal migration to family resettlement and from temporary habitation to permanent residence [1]. The 2010 census estimated that there were 236 million migrants in China, of which 35.81 million were children under the age of 18 [1,2]. Of these, 17.54 million were children ranging in age from 12 to 17 years old [1]. In 2010, the 1.06 million migrant children in the city of Beijing alone accounted for 36.28% of the city’s total population of children [1].

Urban residents in China obtain household registration (HuKou) credentials, which associates them with a particular town or city. The household registration system was designed to control rural-urban mobility for economic and political purposes, but it also permits residents access to a variety of amenities provided by their town or city. Because migrant children in cities do not have a local household registration, they are considered temporary residents and are not granted equal access to education, medical care and other social services in the cities where they live. Large numbers of migrant children are thus excluded from cities’ public schools and must enroll in privately operated *migrant schools*, which were established as informal schools that served the educational needs only of migrant children in cities [3]. Compared with public schools, migrant school are of significantly lower quality because they must depend on very limited private support. Recent policies have sought to address this education problem involving migrant children, and the government has thus begun to subsidize some migrant schools and/or has begun to require public schools to accommodate migrant children. However, even if migrant children are able to receive an education in public schools, they remain ineligible for the high-school entrance examination in the host city and must return to their hometowns to take it. Moreover, delays in policy implementation have perpetuated a harsh situation and have disadvantaged migrant children [4].

A number of studies in China have demonstrated that migrant children are at high risk to exhibit delays in adaptive functioning, including social, emotional, and behavioral problems [5–7]. One explanation for migrant children’s maladjustment is the discrimination they experience based on their HuKou and the circumstances of their rural-urban migration; such discrimination constitutes a potential threat to the adjustment of these children [7,8]. Previous studies [9–11] have found that the probability of experiencing discrimination is increased for adolescents because they typically spend more social time outside of the home. Thus, scholars have called for more studies examining the effects of discrimination on psychosocial adjustment during adolescence, in particular [10,12].

Although perceived discrimination has been linked to negative outcomes for adolescents, some studies have found that there is considerable variation in the adaptation of Chinese migrant adolescents; some migrant adolescents do not experience adaptive problems [6], and some even show positive development [7,13]. In other words, perceived discrimination does not always result in negative outcomes for Chinese migrant adolescents [7,14].

Therefore, it is important to identify the factors (e.g., group identity) that may buffer the negative effects of discrimination. However, research related to the processes that may contribute to resilient adaptation in these highly disadvantaged Chinese migrant children is extremely rare. Grounded in the risk and resilience framework, which posits that individuals may have access to resources that can help them overcome the risks associated with adverse experiences [15,16], the purpose of this study was to examine whether group identity might minimize the negative effects of perceived discrimination on Chinese migrant adolescents’ psychological well-being. Further, we aimed to examine the type of school attended as a moderator of the associations among perceived discrimination, group identity, and psychological well-being.

### Perceived Discrimination and Psychological Well-being

Perceived discrimination has been defined as the subjective experience of being treated unfairly relative to others in everyday experience [17] and is a type of social stress experienced mainly by low status groups based on their group membership (e.g., ethnicity, gender, socio-economic status, and–in this instance–HuKou) that separates an individual from the dominant group. Previous studies have documented that experiences of discrimination are salient among Chinese migrant adolescents based on their HuKou. For example, migrant adolescents have reported that chronic incidents of discrimination were the most stressful experiences in their lives [7]. In other studies, 75.5% of migrant adolescents reported that they were the victims of discrimination [6,18], and 75.7% of migrant children (including adolescents) reported experiencing discrimination–including sarcasm or insults–in their daily lives [19].

Theoretical formulations propose that perceived discrimination increases the likelihood of negative developmental outcomes and reduces the occurrence of positive developmental outcomes among minority groups [20,21]. In particular, research on discrimination among Chinese migrant adolescents has demonstrated the existence of such a relationship. For example, perceived discrimination has been linked to increased symptoms of depression [6,22], increased symptoms of social anxiety [6], lower life satisfaction levels [7], and lower self-esteem [10,23]. Longitudinal research has also shown that perceptions of discrimination influence subsequent loneliness [24]. Taken together, these findings suggest that perceived discrimination could be linked to diminished well-being among Chinese migrant adolescents.

Previous studies have shown that there are two components of psychological well-being: personal well-being and collective well-being [25,26]. The indicators of personal well-being mainly include self-esteem, life satisfaction, and mental health symptoms (e.g., depressive moods or anxiety), whereas the assessment of collective well-being mainly involves the use of a scale measuring collective self-esteem [25–30]. Importantly, much of the research on the consequences of perceived discrimination has focused on personal well-being (e.g., personal self-esteem), and few studies have examined the association between perceived discrimination and collective well-being (e.g., collective self-esteem). Consequently, additional research is required that examines the relationships between Chinese migrant adolescents’ perceived discrimination and the indices of both personal and collective well-being, and whether these relationships are moderated by group identity. In the present study, we selected personal self-esteem and life satisfaction as indicators for personal well-being, and we selected collective self-esteem as the indicator for collective well-being.

### Group Identity as a Protective Factor

The risk and resilience framework suggests that individuals with certain strengths will either not be negatively affected by risk or will be affected to a lesser degree than those who are not characterized by these strengths [16]. Consistent with this framework, scholars have suggested that culturally specific mechanisms may help protect minority children from the negative effects of culturally informed stressors, such as perceived discrimination [31]. For example, Phinney [32] argued that group identity can serve as a culturally salient and developmentally relevant factor that enables individuals to be resilient when encountering discrimination. Moreover, group identity is a multidimensional, dynamic construct [33–35]. Recent studies [36–38] have found that the individual components of group identity were distinctly related to adolescent outcomes, and scholars have encouraged researchers to examine the components of group identity independently.

The present study focuses on an affective component of group identity, known as group identity affirmation and belonging (GIAB), which involves positive feelings for–and attachment to–one’s group [39]. We focus our attention on GIAB based largely on notions developed in social identity theory. According to social identity theory [40], those individuals with positive feelings of belonging to a group will remain strongly committed to that group and feel good about their group membership. Those positive feelings may enhance self-concept and help counteract the negative consequences of outside threats. Thus, individuals with a strong sense of GIAB may be protected against the negative effects of perceived discrimination. Moreover, researchers have suggested that this sense of affirmation and belonging to one’s group is a critical dimension of group identity that may play a key role in maintaining psychological health [41] and most closely approximates the social identity component of group identification [42].

Several empirical studies on ethnic minorities have supported this buffering hypothesis regarding the role of GIAB in the link between perceived discrimination and adjustment outcomes. For example, Umaña-Taylor, Wong, Gonzales, & Dumka [43] found that ethnic identity affirmation acted as a significant protective factor by buffering the negative impact of discrimination on the externalizing behaviors of male adolescents of Mexican origin. Eccles, Wong, & Peck [41] found that strong and positive feelings in connection with one’s ethnic group reduced the magnitude of the association between events of racial discrimination and declines in both academic self-concepts and school achievement. Similarly, research involving Navajo adolescents [44] found an increasingly positive relation between events of discrimination and self-esteem as female adolescents’ sense of affirmation of and belonging to Navajo culture increased. Finally, Greene, Way, & Pahl [45] found that self-esteem among Black, Latino, and Asian-American adolescents was less affected by the perceived discrimination of their peers when these adolescents felt a strong sense of affirmation of and belonging to their ethnic group, compared with their peers who exhibited low levels of affirmation and belonging. Together, these studies support the notion of the protective function that GIAB can serve for minority group members facing discrimination. However, so far as we have been able to find, there is no such prior research on the particular problems of Chinese migrant adolescents and perceived discrimination in the extant literature.

For members of low status groups, group boundary permeability is an important determinant of group identification [46,47], and the effect of group identity in the association between perceived discrimination and psychological well-being appears to be based on the likelihood of individual mobility [48]. Previous studies on the buffering hypothesis of group identity have mainly focused on group membership that is relatively enduring and permanent (e.g., race or ethnicity). By contrast, one of the salient qualities of Chinese migrant children consists of the permeability of the group’s boundaries. Migrant children are not distinguished from non-migrant children by physical appearance, as is typically the case with ethnic or racial groups that experience discrimination. Chinese migrant children’s membership in the migrant group depends entirely on an administrative designation and current residence location. Migrant children become migrant children only when they move from a rural area to an urban area and become non-migrant immediately upon moving away from the urban area. Thus, the permeability of these children’s membership is remarkable compared with other low status groups, and is particularly salient for migrant adolescents. After middle school, migrant adolescents have little educational opportunity in the city and must return to their rural hometowns to take the high-school entrance examination, and migrant discrimination disappears when the adolescent returns to his or her HuKou area. No research to date has examined the protective function of group identity for Chinese rural-urban migrant adolescents’ personal and collective well-being. Thus, we believe that the present study may make a significant contribution to the literature on discrimination by extending the understanding of the protective effects of GIAB to a low-status group in which membership is relatively temporary with boundaries that are highly permeable.

### Moderating Role of the Type of School

In China, there are two types of schools that serve the educational needs of migrant adolescents in cities: public schools and privately operated migrant schools. Understanding the differences in the types of schools in the discrimination-adjustment link is critical for understanding which subgroups may be more affected by discriminatory treatment and has profound implications for prevention and intervention programs with respect to Chinese migrant adolescents.

Prior studies indicate that Chinese migrant adolescents in public school report lower levels of perceived discrimination and higher levels of personal self-esteem, life satisfaction, and collective self-esteem than reported by Chinese migrant adolescents in migrant school [6,7,23]. However, it is unclear whether perceived discrimination is less or more negatively linked to the psychological well-being of migrant adolescents in public schools, compared with those in migrant schools. Given that the type of school is an important determinant of Chinese migrant children’s academic and psychological adjustment [3,6,7], the present study aimed to examine the type of school (public vs. migrant) as a moderator of the associations among perceived discrimination, GIAB, and well-being.

### The Present Study

On the basis of previous research regarding perceived discrimination, we examined three main research questions. First, we assessed the relationship between perceived discrimination and three indicators of psychological well-being: self-esteem, life satisfaction, and collective self-esteem. Previous studies have examined only the effects of perceived discrimination on migrant adolescents’ personal well-being [6,22]; however, we also include collective well-being in our study. Based on previous conceptual and empirical work, we hypothesized that perceived discrimination would be associated with lower levels of personal self-esteem, life satisfaction, and collective self-esteem.

Second, we explored the potential moderating roles of GIAB on the relationships between perceived discrimination and psychological well-being. Consistent with the risk and resilience framework and with prior work on identity [16,32,37,43], we also hypothesized that GIAB would moderate the association between perceived discrimination and each of these indices of psychological well-being. We further expected that the association would be significantly weaker among adolescents who exhibited higher levels of GIAB.

Finally, prior research suggests that public school’s migrant adolescents and migrant school’s adolescents might differ in their experiences of both perceived discrimination and psychological well-being [6,23]. Therefore, we tested whether the type of school (public school or migrant school) moderated the relationships of migrant adolescents’ perceived discrimination, GIAB, and psychological well-being. Although previous research has found mean level differences based on the type of school among the variables of interest in the present study, we did not expect the overall process (e.g., the associations among these variables) to differ across school types. We controlled for age, gender and length of residence in the city in our analyses because prior research has found differences with respect to age, gender and length of residence in reports of migrant adolescents’ psychological well-being [6,7,49].

## Method

### Participants and Procedures

Participants in our sample included 798 Chinese migrant adolescents in the fifth through eighth grades from three migrant schools (50.8%) and four public schools (49.2%) in urban areas of Beijing. The mean age of the sample was 13.10 years (SD = 1.48); 52.1% were boys and 47.9% were girls. All adolescents were born outside Beijing city and reported living in Beijing for an average of 5.71 years (SD = 3.40) since they had migrated from a rural area. Participants reported the following educational levels for their parents: 72.2% of their fathers had less than a high school education, 22.5% had completed high school, and 5.3% had received some level of college education; in addition, 82% of their mothers had less than a high school education, 15.1% had completed high school, and 2.9% had received some level of college education. The data are available from S1 Dataset.

This study was part of a larger investigation of psychological adjustment among left-behind and migrant children in China [50]. During the process of recruiting participants, a number of public schools and private schools (i.e., migrant schools) were contacted and invited to participate in the study. Seven schools were selected on the basis of the principals’ willingness to participate in the study. Approval was obtained from the school district. We recruited all the migrant adolescents in the fifth through eighth grade at seven schools as the participants in the study. Written informed consent was obtained from the parents of participating students. Students and their parents were given a description of the purpose of the study and the procedures planned. The migrant adolescents were reminded that their participation was voluntary and that the results from their questionnaires would remain confidential. All the participants were of the Han nationality, which is the predominant ethnic group in China and accounts for more than 90% of the population. None of the participants had an observable physical or developmental disability. The study protocol was approved by the Institutional Review Board of Beijing Normal University and complied with the ethical standards for research involving human subjects.

The data for this study were collected during the 2010–2011 period. Data collection was conducted in each of the classrooms during regular school hours. The series of questionnaires required approximately 45 minutes to complete. Three research assistants administered the surveys, answered questions, and monitored participants’ progress. One assistant also read each question aloud as the participants followed along to ensure that students understood the content of the items.

### Measures

#### Demographic form

Participants completed a brief demographic form that provided background information on age, gender, school type, ethnic group, length of residence in the city, and parental education.

#### Perceived discrimination

The 20-item perceived discrimination scale for Chinese migrant adolescents [10] was used to assess perceived discrimination at school and outside school with no time frame specified. Participants rated each item on a 5-point Likert scale ranging from 1 (*strongly disagree*) to 5 (*strongly agree*). Sample items included, “I am excluded because I am a migrant child” and “I am teased because I am a migrant child”; mean scores were used in the analyses, with higher scores indicating greater perceived discrimination. This scale has been shown to be reliable and valid for use with Chinese migrant adolescents [8]. For the present sample, Cronbach’s alpha was .88.

#### Group identity affirmation and belonging (GIAB)

We used the Multigroup Ethnic Identity Measure [51] to measure group identity. The 12-item measure consists of statements about feelings regarding–and reactions to–one’s ethnicity. The measure has two subscales: (a) affirmation, belonging, and commitment and (b) ethnic identity search. Only the 7-item affirmation, belonging, and commitment subscale was used in the present study. Each item was rated on a 4-point Likert scale ranging from 1 (*strongly disagree*) to 4 (*strongly agree*), participants rated the degree to which they agreed with each statement, with higher values indicating more GIAB. A sample item included, “I am happy that I am a member of the group I belong to”; mean scores were used in the analyses. The measure has been used previously with Chinese migrant children with a Cronbach’s alpha above .80 [52]. For the present sample, Cronbach’s alpha was .78.

#### Personal well-being

Following previous studies [26,27], we assessed personal well-being using two measures. The Chinese version of the Rosenberg Self-Esteem Scale [53] is a validated measure of global personal self-esteem. The 10-items use Likert rating scales in assessing statements relating to self-esteem (e.g., “I feel that I have a number of good qualities”). Ratings options ranged from 1 (*strongly disagree*) to 6 (*strongly agree*); mean scores were used in the analyses. Among other samples of Chinese migrant adolescents, the scale has demonstrated adequate reliability with a coefficient alpha of 0.83 [54]. The Cronbach’s alpha for the present sample was .80. Personal well-being was also measured by the Students’ Life Satisfaction Scale [55], which measures satisfaction with life as a whole (e.g., “I have what I want in life”). The seven items are rated using Likert scales with responses ranging from 1 (*strongly disagree*) to 5 (*strongly agree*); mean scores across all items were used in the analyses. For the present sample, Cronbach’s alpha was .72.

#### Collective well-being

We measured collective well-being using two subscales from the Collective Self-Esteem (CSE) scale [56]. Two 4-item subscales measured (a) private collective esteem (how positively individuals judge their ethnic group, e.g., “In general, I am glad to be a member of the migrant children group”) and (b) membership self-esteem (how good or worthy a member they are of their ethnic group, e.g., “I am a worthy member of the group of migrant children”). Participants rated items on a 6-point scale ranging from 1 (*strongly disagree*) to 6 (*strongly agree*); mean scores across all items were used in the analyses. The identity and public subscales from CSE were not included because other studies suggest that identity subscales measure ethnic identification as opposed to ethnic self-esteem. Additionally, the public subscale potentially overlaps with perceptions of discrimination [25]. Prior research has demonstrated adequate reliability in Chinese adolescents’ samples with Cronbach’s alpha above 0.80 [57]. For the present sample, Cronbach’s alpha for the overall scale measured .75.

### Plan of Analysis

Analyses were conducted using IBM SPSS Statistics 20. We began by calculating the preliminary analyses, including descriptive statistics, correlations, and *t*-tests of type of school and gender differences for key variables. Next, a series of hierarchical regression analyses was used to examine the moderating effects of GIAB and the types of schools on the relation between perceived discrimination and the indices of psychological well-being (self-esteem, life satisfaction, and collective self-esteem). For each hierarchical regression, age, gender and length of residence in the city were entered as control variables in the first step. We entered perceived discrimination, GIAB, and the type of school as control variables in the second step. We added the two-way interaction terms in the third step. Finally, in the fourth step, we inserted the three-way interaction term. All continuous variables were centered and the variables gender (0 = girls) and school type (0 = public school) were dummy coded to reduce problems of multicollinearity. Tests were run to examine variance inflation factors (VIF) values, which are indicators of multicollinearity. None of the results measured higher than 1.6, indicating that multicollinearity between variables was at an acceptable level [58].

## Results

### Descriptive Analyses

Table 1 summarizes the means, standard deviations, and zero-order correlations for the study variables. Statistical analysis was carried out on all available data without replacement. All the psychological well-being variables correlated negatively with perceived discrimination and positively with GIAB. Perceived discrimination was negatively correlated with GIAB. Age was positively associated with perceived discrimination and negatively associated with life satisfaction and collective self-esteem. Length of residence in the city was positively associated with self-esteem and life satisfaction and negatively with perceived discrimination. *T*-tests were conducted to assess the type of school and gender differences in the variables of interest (Table 2), and the results indicated that adolescents in migrant schools perceived more discrimination and had lower levels of self-esteem, life satisfaction, and collective self-esteem than migrant adolescents in public schools. In addition, boys perceived more discrimination and had lower levels of collective self-esteem levels than girls.

**Table 1:** Correlations, means, and standard deviations for the study variables.

Variable	1	2	3	4	5	6	7
1. Self-esteem	—						
2. Life satisfaction	.38^***^	—					
3. Collective self-esteem	.55^***^	.29^***^	—				
4. Perceived discrimination	−.43^***^	−.36^***^	−.43^***^	—			
5. GIAB	.35^***^	.19^***^	.55^***^	−.23^***^	—		
6. Age	−.01	−.12^***^	−.08^*^	.14^**^	−.02	—	
7. Length of residence in the city	.11^**^	.12^***^	.04	−.10^**^	.05	.01	—
Mean	3.72	3.07	4.71	2.37	4.48	13.10	5.71
SD	.66	.73	.88	.69	.98	1.48	3.40

*Note*. GIAB = group identity affirmation and belonging.

* *p* < .05

** *p* < .01; and

*** *p* < .001.

**Table 2:** Means and standard deviations of key variables by school type and gender.

	School type			Gender		
	Migrant	Public		Girls	Boys	
	Mean (SD)	Mean (SD)	*t*	Mean (SD)	Mean (SD)	*t*
Self-esteem	3.60(.58)	3.86(.71)	5.42^***^	3.76(.64)	3.69(.68)	1.56
Life satisfaction	2.92(.68)	3.23(.76)	6.03^***^	3.03(.75)	3.11(.72)	−1.49
Collective self-esteem	4.63(.83)	4.79(.93)	2.46^*^	4.81(.82)	4.61(.93)	3.32^***^
Perceived discrimination	2.56(.64)	2.16(.68)	−8.37^***^	2.30(.64)	2.44(.73)	−2.84^**^
GIAB	4.47(.94)	4.49(1.02)	.22	4.53(.93)	4.43(1.02)	1.45

*Note*. GIAB = group identity affirmation and belonging; migrant = migrant school; public = public school.

* *p* < .05

** *p* < .01; and

*** *p* < .001.

### Predicting Personal Well-being

**Fig 1:**
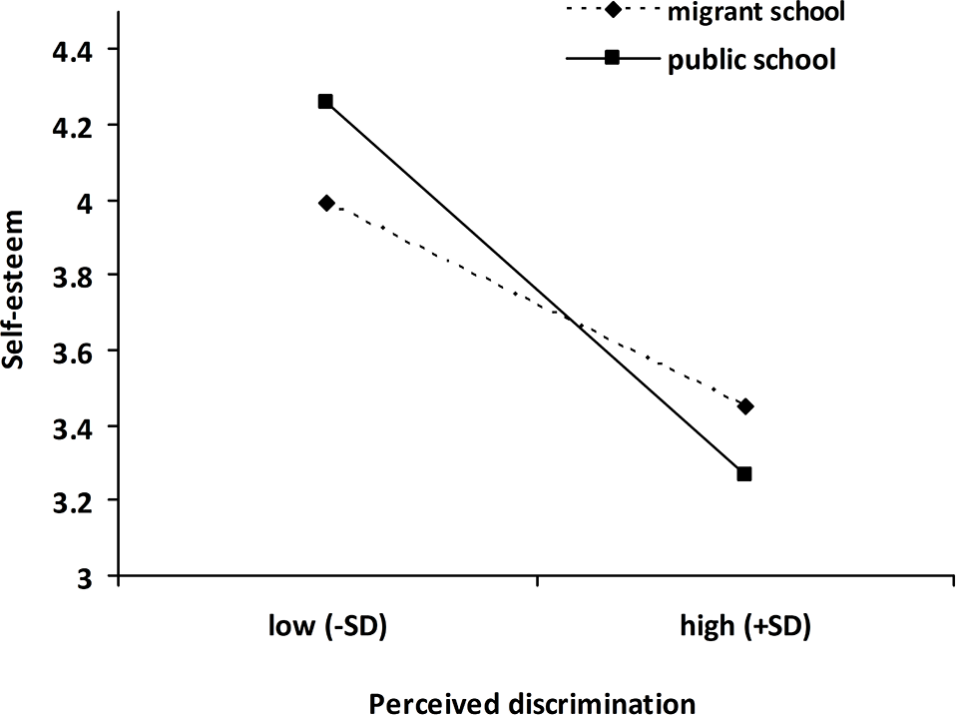
School type as a moderator of the relationship between perceived discrimination and self-esteem. Low/high perceived discrimination represents ± 1 *SD* of the mean.

**Fig 2:**
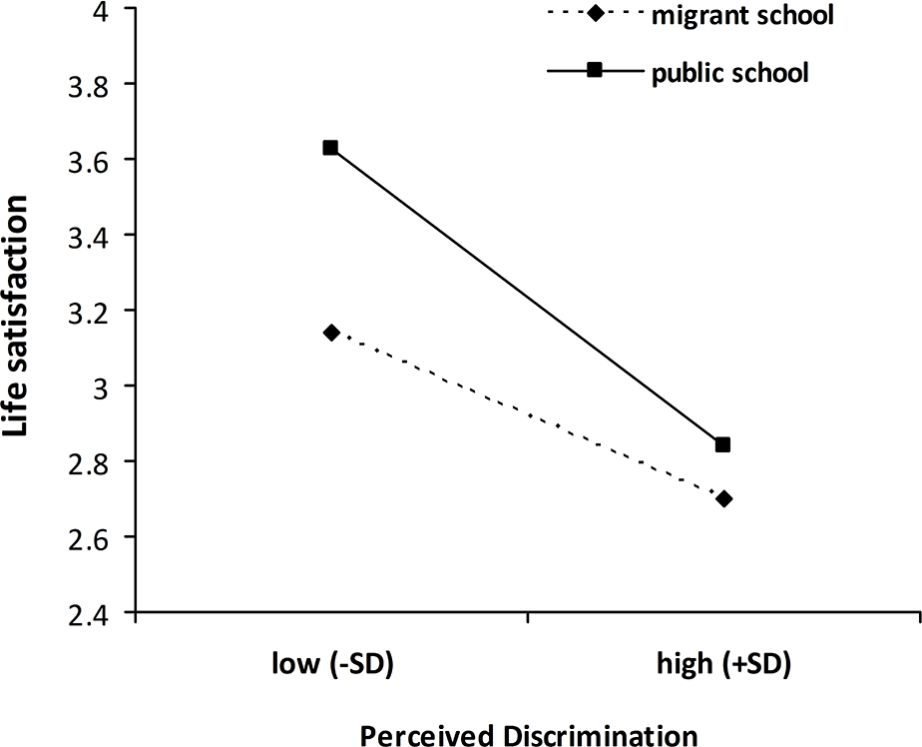
School type as a moderator of the relationship between perceived discrimination and life satisfaction. Low/high perceived discrimination represents ± 1 *SD* of the mean.

Perceived discrimination was significantly associated with lower levels of self-esteem and life satisfaction, after controlling for age, gender and length of residence in the city (Table 3). GIAB was associated with higher levels of self-esteem and life satisfaction. The interaction term (perceived discrimination × school type) was significant for self-esteem and life satisfaction. Moderation analyses were conducted using the PROCESS tool in SPSS 20 [59]. As shown in Figs 1 and 2, higher levels of perceived discrimination were associated with lower levels of life satisfaction and self-esteem for migrant adolescents in both migrant school (self-esteem, *B* = −.27, *t* = -5.82, *p* < 0.001; life satisfaction, *B* = −.24, *t* = -4.59, *p* < 0.001) and public school (self-esteem, *B* = −.54, *t* = -11.14, *p* < 0.001; life satisfaction, *B* = −.46, *t* = -8.70, *p* < 0.001). This relationship, however, was more pronounced among migrant adolescents in public school. GIAB did not moderate the relationship between perceived discrimination and either outcome, i.e., life satisfaction and self-esteem (see Table 3).

**Table 3:** Hierarchical regression analyses testing the moderating effects of group identity and the type of school in the relationship of perceived discrimination to personal and collective well-being.

	Life satisfaction				Self-esteem				Collective self-esteem			
Variable	*β*	*B*	SE	*△R*^*2*^	*β*	*B*	SE	*△R*^*2*^	*β*	*B*	SE	*△R*^*2*^
Step 1 (control variables)				.03^***^				.03^***^				.03^***^
Age	−.10^**^	−.05	.02		.01	.01	.02		−.07	−.04	.02	
Gender	.01	.01	.06		−.09^*^	−.12	.05		−.14^***^	−.24	.07	
Length	.13^***^	.03	.01		.15^***^	.03	.01		.07	.02	.01	
Step 2				.14^***^				.23^***^				.38^***^
PD	−.31^***^	−.33	.04		−.35^***^	−.34	.04		−.30^***^	−.38	.04	
GIAB	.11^**^	.08	.03		.25^***^	.17	.02		.47^***^	.42	.03	
ST	−.10^*^	−.15	.06		−.06	−.09	.05		−.02	−.04	.06	
Step 3				.01^*^				.01^*^				.02^***^
PD × GIAB	.05	.05	.04		.05	.04	.03		.13^***^	.15	.04	
PD × ST	.09^*^	.19	.08		.09^**^	.19	.07		.09^**^	.23	.08	
GIAB × ST	−.04	−.06	.06		−.02	−.02	.05		−.03	−.05	.06	
Step 4				.00				.00				.00
PD × GIAB × ST	.01	.02	.07		−.02	−.04	.06		.02	.06	.07	

Note. 0 = girls; 1 = boys; 0 = public school; 1 = migrant school; Length = Length of residence in the city; ST = school type; PD = perceived discrimination; and GIAB = group identity affirmation and belonging.

* *p* < .05

** *p* < .01; and

*** *p* < .001.

### Predicting Collective Well-being

**Fig 3:**
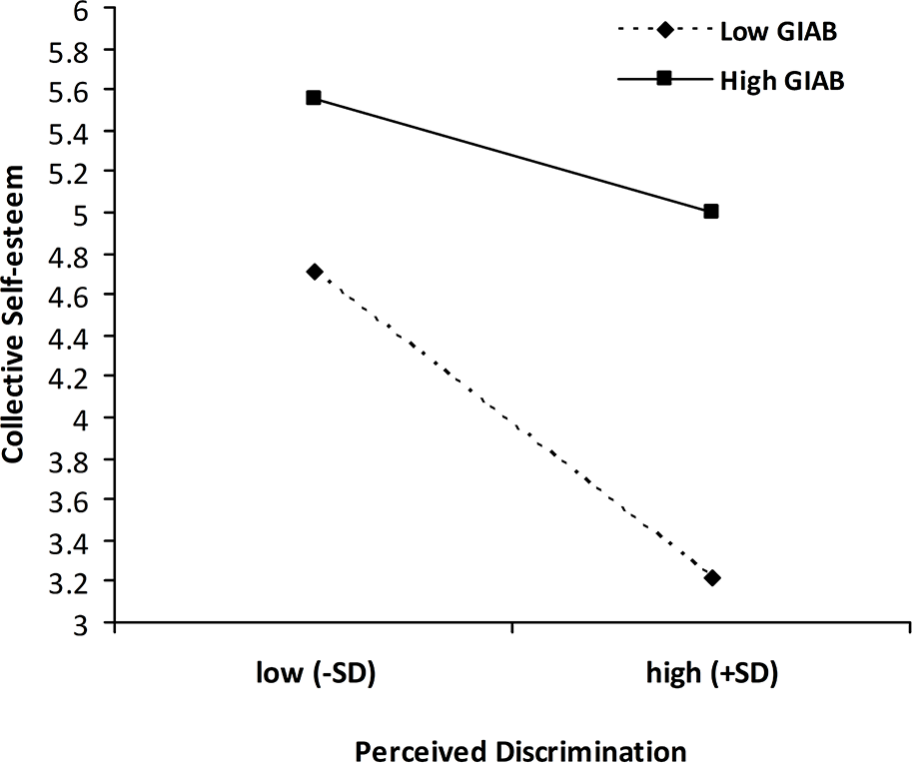
Moderation of the association between perceived discrimination and collective self-esteem by group identity affirmation and belonging (GIAB). Low/high perceived discrimination and low/high GIAB represents ± 1 *SD* of the mean.

**Fig 4:**
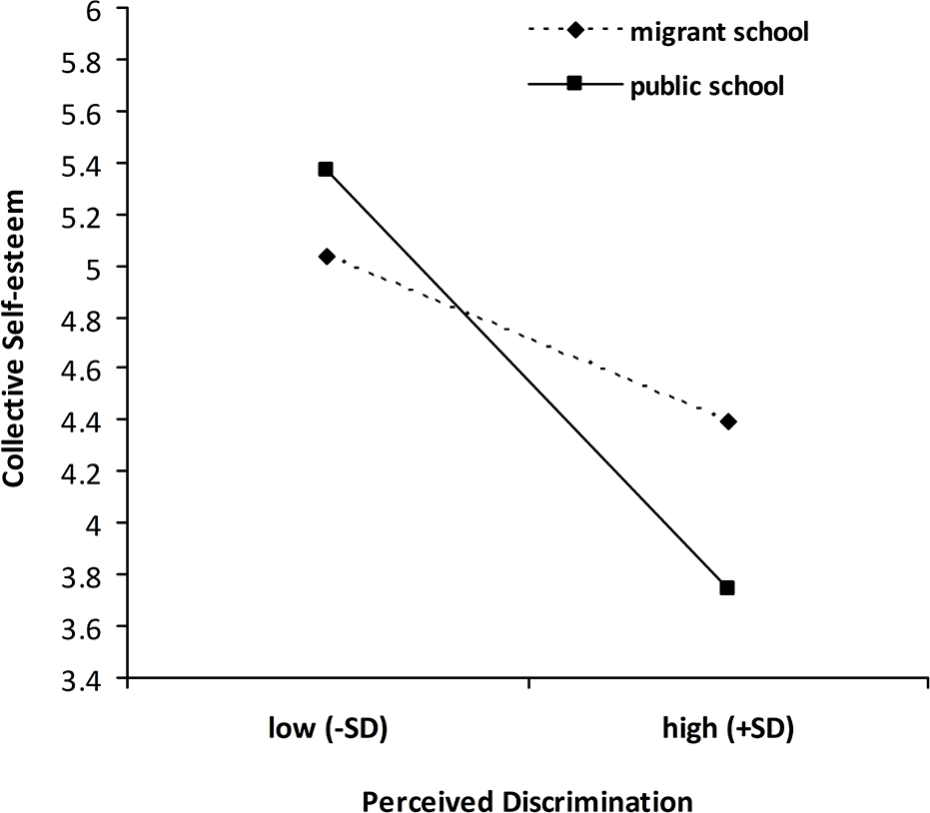
School type as a moderator of the relationship between perceived discrimination and collective self-esteem. Low/high perceived discrimination represents ± 1 *SD* of the mean.

The main effects of perceived discrimination and GIAB were found for collective self-esteem (Table 3). Perceived discrimination was significantly associated with lower levels of collective self-esteem, whereas GIAB was significantly associated with higher levels of collective self-esteem. Two significant two-way interactions (perceived discrimination × GIAB and perceived discrimination × school type) were found for collective self-esteem. Fig 3 shows that, although the simple slopes for migrant adolescents who reported both high and low levels of GIAB were significant, the negative effects of perceived discrimination on collective self-esteem appeared stronger for migrant adolescents with lower levels of GIAB (*B* = −.56, *t* = -11.46, *p* < 0.001) compared with those with higher levels of GIAB (*B* = −.25, *t* = -5.00, *p* < 0.001). In addition, migrant adolescents in public school (*B* = −.79, *t* = -12.46, *p* < 0.001) reported lower collective self-esteem when they reported higher perceived discrimination than adolescents in migrant school reported (*B* = −.36, *t* = -5.86, *p* < 0.001; see Fig 4).

## Discussion

This study aimed to extend the scope of the relationships between perceived discrimination and well-being among Chinese migrant adolescents by examining the potential protective roles of adolescents’ group identity. Our findings suggest that the deleterious effects of perceived discrimination on migrant adolescents’ personal and collective well-being may be particularly salient for those migrant adolescents in public school. Importantly–and consistent with the risk and resilience framework [15,16]–we found that migrant adolescents’ positive feelings about their group background (i.e., GIAB) performed a protective function with respect to collective well-being by minimizing the negative effects of perceived discrimination.

### Relationships between Perceived Discrimination and Well-Being

Our findings corroborated the theoretical formulations in that we found that discrimination was related to adolescents’ psychological functioning [20]. Consistent with the study hypothesis and previous research on relationships between perceived discrimination and psychological well-being [6,23], our findings offer additional evidence regarding the negative effects of discrimination on the well-being of Chinese migrant adolescents. This finding is particularly important because it focuses on a group with relatively permeable membership boundaries. For these adolescents that migrated from rural to urban locations with their parents, higher levels of perceived discrimination were significantly associated with lower levels of self-esteem, life satisfaction, and collective self-esteem. These significant associations were evident not only at the bivariate level but also at the multivariate level, after controlling for age, gender and length of residence in the city. Our examination of three indicators of psychological well-being provided a more holistic perspective on the impact that discrimination may have on migrant adolescents’ well-being. The next important step to be taken by future research is to examine the longitudinal and reciprocal relationships between discrimination and well-being.

### Moderating Role of GIAB

Previous studies on the buffering hypothesis of group identity have primarily focused on minority groups whose group membership is relatively enduring and permanent (such as race or ethnicity). No research to date has examined the protective function of group identity among Chinese rural-urban migrant adolescents whose lower status group membership is relatively temporary. Providing some resilience in the face of the stress of discrimination, we found that GIAB acts as a positive resource in migrant adolescents’ lives. Direct effects were found whereby GIAB was associated with three indicators of well-being (i.e., self-esteem, life satisfaction, collective self-esteem). These results are congruent with previous research documenting the positive links between group identity and psychological well-being [25,52]. Moreover, GIAB buffered the negative effects of perceived discrimination on the collective well-being of migrant adolescents. For migrant adolescents reporting higher levels of GIAB, the negative effect of perceived discrimination on collective self-esteem appeared weaker, compared with those who reported lower levels of GIAB. These findings were in line with our predictions and underscore the importance of group identity in Chinese migrant adolescents who are coping with discrimination. Future prevention and intervention efforts may focus on increasing migrant adolescents’ sense of attachment to their group. Our findings extend prior research by focusing on collective well-being as an outcome variable, whereas prior research [6,22,23] has focused largely on migrant adolescents’ personal well-being outcomes.

Although GIAB emerged as a protective buffer against the potential negative effects of perceived discrimination on migrant adolescents’ collective well-being, we were surprised to find, contrary to our expectations, that the relationship between perceived discrimination and personal well-being did not provide support for GIAB as a protective factor in this sample. In other words, we found no evidence that GIAB moderated the relationship between migrant adolescents’ perceived discrimination and personal well-being (i.e., self-esteem and life satisfaction). This finding seems inconsistent with prior empirical literature regarding the role of group cultural resources: Identification with one’s group culture should provide extra coping resources and should serve to mitigate the stress of discrimination and accompanying adjustment problems [37,44,45]. Notably, several prior studies have also reported that identification with group culture does not produce moderating effects [60,61]. For example, Toomey et al. [61] found that ethnic identity buffered the link between discrimination and externalizing risky behavior but not between discrimination and internalizing symptoms or self-esteem among adolescent mothers of Mexican origin. One possibility is that other processes or mechanisms (e.g., coping strategies involving distraction) may be more salient for disrupting the link between discrimination and internalizing symptoms [61], which underscores the importance of identifying other potential protective factors for Chinese migrant adolescents’ self-esteem and life satisfaction.

Another possibility is that cultural factors such as GIAB may play a mediating and not a moderating role in the relationship between discrimination and internalization. For example, previous research with Mexican-American adolescents [62] provided support for Mexican-American values as a risk reducer–rather than as a protective factor–to counteract both the effects of discrimination on academic outcomes and the effects of internalizing discrimination. Recently, Shen et al. [7] found that GIAB exerted a partial mediating effect on the relationship between discrimination and internalizing problems in their study of Chinese migrant children. Future research should be expanded to examine the multiple mechanisms of protective processes in understanding the resilience of Chinese migrant adolescents.

In addition, the adolescent developmental period may be an important factor to consider in understanding the absence of the moderation effects of GIAB, given previous empirical work with older adolescents that suggests that GIAB buffered the link between discrimination and self-esteem [44,45]. The individuals in the sample in the present study were early adolescents, which is the period when these individuals are still exploring and discovering what it means to be a member of a migrant group. These adolescents thus remain in the early stages of understanding this abstract concept and not yet able to reap its protective effect. As adolescents progress through middle and late adolescence, GIAB may serve a stronger and more significant protective function by buffering the negative impact of discrimination on migrant adolescents’ self-esteem and life satisfaction. Future research should examine these associations among older migrant adolescents.

### Differences in the Type of School and Psychological Well-being

Turning to variability by the type of school attended, although previous studies have focused on the differences in the type of school in the adjustment outcomes of Chinese migrant children [6,7,23], many questions remain unanswered regarding whether the relationship between perceived discrimination and psychological well-being varies with the type of school attended by an adolescent. Therefore, in the current study, we test the differences in the type of school in the associations among perceived discrimination, GIAB, and well-being.

In so doing, we found that although migrant adolescents in migrant school perceived more discrimination than those in public school, the negative effects of perceived discrimination on personal and collective well-being were more pronounced among migrant adolescents in public school, which suggests that the psychological well-being of public school’s migrant adolescents may be more vulnerable to perceived discrimination. Notably, this finding is consistent with a prior study involving another sample of Chinese migrant children [63] that showed that perceived discrimination had a stronger negative effect on children in public school than on children in migrant school. It may be that the migrant adolescents in public school have higher levels of sense of belonging to the city and more resident identification than their migrant school counterparts [64] and are therefore more likely to believe that they belong in the city. Therefore, perceived discrimination may be more likely to affect public school’s migrant adolescents because they will feel that other students reject their membership (i.e., belonging in the city) and that they do not receive the equal treatment that they expect [63]. Our findings suggest that the type of school is an important variable in understanding the associations between Chinese migrant adolescents’ perceived discrimination and well-being, and that prevention and intervention efforts may need to be developed for specific subgroups of Chinese migrant adolescents who may be more vulnerable to events of discrimination.

### Caveats and future directions

Although the findings of this study provide a starting point for examining the specific conditions under which the relationships between Chinese migrant adolescents’ perceived discrimination and psychological well-being are moderated, we urge some caution in interpreting our results. First, the sample is cross-sectional; thus, although the results suggest interesting trends, the possibility of cohort effects remain. Longitudinal research is required to replicate our findings and to examine how the relationship between perceived discrimination, group identity, and psychological well-being play out over time. For example, one set of unanswered questions is whether there are similar patterns during middle through late adolescence, and if so, by what mechanisms.

We also note that the measure of perceived discrimination that we used specified no defined time frame. Thus, participants might have been recalling instances of perceived discrimination from any time in their past lives. More precise measures of perceived discrimination might aid in understanding shorter time periods (i.e., daily, monthly, or annually). Developing such measures would be a useful target for future research.

In addition, the permeability of migrant group boundaries also suggests some circumspection. Individuals enter the migrant group when they move to an urban area; similarly, they leave the migrant group by moving back to their rural home. Such events occur over individually determined schedules, and the time frames during which individuals are part of the migrant group thus present some complexity in analyzing any effects, particularly those involving discrimination against members of the group.

Finally, because this study focused on a sample of Chinese migrant adolescents in Beijing, interpretations and generalizations of the current findings must be mindful of context. Studies using a more diverse sample of Chinese migrant adolescents are thus warranted. Additionally, Given that adolescents were nested within seven schools, which were not enough for multilevel modeling [65], we conducted our analyses using hierarchical regression in the present study. Therefore, the inability to account for the fact that the data were nested within schools should be noted.

## Conclusion

The present study adds to the previous literature implying that discrimination is a serious threat for Chinese migrant adolescents and underscores the need to consider GIAB and the type of school as important variables in understanding the role that discrimination plays in the psychological well-being of Chinese migrant adolescents.

This study is consistent with prior empirical research suggesting that perceived discrimination is a stressor for adolescents, although not all adolescents appear to be equally affected [43,27]. Our findings suggest that the negative effects of discrimination on psychological well-being are of greater consequence for migrant adolescents in public school than for migrant adolescents in migrant school. Thus, it is imperative to focus preventive intervention efforts regarding discrimination against migrant adolescents in public schools. Our findings also suggest that high levels of GIAB help protect Chinese migrant adolescents against the impact of perceived discrimination on collective well-being, which underscores the importance of group identity in helping these adolescents’ cope with discrimination. Future prevention and intervention efforts may thus focus on increasing migrant adolescents’ sense of attachment to their group. Finally, although GIAB did not buffer the association between perceived discrimination and personal well-being, the findings of the present study extend research on Chinese migrant adolescents and suggest that further attention should be given to identifying other buffers of discrimination that help protect Chinese migrant adolescents’ personal well-being.

## Supporting Information

S1 Dataset Data of the present study. (XLS)
